# Advances and Deep Insights Into Lactylation: Essential Roles in Cellular Regulation and Disease Association

**DOI:** 10.1111/jcmm.70810

**Published:** 2025-09-22

**Authors:** Tong Pan, Can‐can Du, Ying‐jian Zhang, Zhen‐long Liu

**Affiliations:** ^1^ Department of Sports Medicine Peking University Third Hospital, Institute of Sports Medicine of Peking University Beijing China; ^2^ Beijing Key Laboratory of Sports Injuries Beijing China; ^3^ Engineering Research Center of Sports Trauma Treatment Technology and Devices, Ministry of Education Beijing China; ^4^ Peking University Sixth Hospital Peking University Institute of Mental Health, NHC Key Laboratory of Mental Health (Peking University), National Clinical Research Center for Mental Disorders (Peking University Sixth Hospital) Beijing China

**Keywords:** cellular regulation, disease association, lactate, lactylation, molecular mechanism

## Abstract

Proteins exert biological functions not only depending on abundance but also on regulation. Lactylation is an important novel post‐translational modification (PTM) that can affect protein structure and function, playing a crucial role in signal transduction, gene expression and cellular metabolism. Lactylation participates in the progression of various diseases, such as tumours, heart failure, myocardial infarction, renal fibrosis and Alzheimer's disease. These studies suggest that lactylation may mediate metabolic reprogramming and enhance cellular plasticity, providing new entry points for developing therapeutic approaches. This review introduces the progress on various aspects of lactylation, including mechanisms, regulations and disease associations, aiming to provide valuable insights and inspiration for further exploration of protein modification networks.

**Abbreviations**
α‐MHC: α‐myosin heavy chain
2‐DG: 2‐deoxy‐D‐glucose
4‐CIN: α‐cyano‐4‐hydroxycinnamate
AARS: alany‐tRNA synthetase
ACSL4: long‐chain acyl‐CoA synthetase 4
ACSS2: acetyl‐CoA synthetase 2
AD: Alzheimer's Disease
ALDH: aldehyde dehydrogenase
ALDO: aldolase
ARF1: ADP‐ribosylation factor 1
ARG1: arginase 1
cGAS: cyclic GMP‐AMP synthase
CKD: chronic kidney disease
DCA: sodium dichloroacetate
DML: demethylzeylasteral
eEF1A2: eukaryotic elongation factor 1 alpha 2
EGFR: epidermal growth factor receptor
ENO: enolase
FGF: fibroblast growth factor
Glis1: GLIS family zinc finger 1
GLO: glyoxalase
GSH: glutathione
GTPSCS: GTP‐dependent succinyl‐CoA synthetase
HDAC: histone deacetylase
HK: hexokinase
HMGB1: high mobility group box‐1
HRR: homologous recombination repair
IDH: isocitrate dehydrogenase
IFI16: gamma‐interferon‐inducible protein 16
Ikzf1: IKAROS Family Zinc Finger 1
Kac: lysine acetylation
KAT: lysine acetytransferase
Kce: lysine N‐ε‐carboxyethyl
Kla: lysine lactylation
LDH: lactate dehydrogenase
LGSH: lactoylglutathione
LPS: lipopolysaccharide
LRP1: low‐density lipoprotein receptor‐related protein 1
MCT: monocarboxylate transporter
MGO: methylglyoxal
MMP: matrix metalloproteinase
MREE11: meiotic recombination 11 homologue A
NAM: nicotinamide
NBS1: Nijmegen breakage syndrome protein 1
OXA: sodium oxalate
OXPHOS: oxidative phosphorylation
PBMC: peripheral blood mononuclear cell
PCK: phosphoenolpyruvate carboxykinase
PFK: phosphofructokinase
PGK1: phosphoglycerate kinase 1
PKM: pyruvate kinase
PTM: post translational modification
ROS: reactive oxygen species
SIRT: sirtuin
SNAP91: synaptic protein 91
TCA cycle: tricarboxylic acid cycle
TGF‐β1: transforming growth factor‐β1
TNF: tumour necrosis factor
TRAP1: tumour necrosis factor receptor‐associated protein 1
VEGF: vascular endothelial growth factor
VSMC: vascular smooth muscle cell
XRCC1: X‐ray repair complementing defective repair in Chinese hamster cells 1
YAP: yes‐associated protein
YnLac: sodium‐(S)‐2‐hydroxypent‐4‐ynoate
YTHDF2: YTH N6‐methyladenosine RNA‐binding protein 2
ZGA: zygotic genome activation

## 1 Introduction

As the direct source of lactylation, lactate has long been regarded as by‐product of glycolysis, or even metabolic waste. However, with in‐depth research, people have gradually reassessed lactate and found it actively participates in energy metabolism, tumour microenvironment, signal transduction and immune regulation. In energy metabolism, lactate can enter the tricarboxylic acid (TCA) cycle, participate in the ‘muscle‐liver‐muscle’ lactate Cori cycle [1] and the ‘astrocyte‐neuron lactate shuttle’ [2]. In tumour microenvironment, lactate accumulation caused by the Warburg effect is a fundamental characteristic [3]. The lactate concentration can reach 40 mmol/L, while the normal blood lactate concentration is only 2 mmol/L [4]. Perhaps the Warburg effect can provide survival advantages for tumours. For example, it can provide a three—carbon lactate carbon source for biosynthesis, avoid accidental killing caused by reactive oxygen species (ROS) generated by oxidative phosphorylation (OXPHOS) [5], the microenvironment of lactic acidosis can change the tumour‐stroma interface to enhance the ability of invasion and spread [6], and even induce the M2 polarisation of tumour associated macrophages (TAMs), leading to immune evasion [7].

In addition, lactate also mediates a novel post‐translational modification: lysine lactylation (Kla). In 2019, Zhang et al. [8] first reported that histone lactylation can affect chromatin status and activate gene expression. This means that lactate plays a dual regulatory role in epigenetic regulation and metabolic reprogramming, converting the metabolic signal into a DNA transcription signal. Subsequent studies have shown that lactylation can affect protein localisation, regulate protein activity, influence protein stability and affect protein–protein interactions. Lactylation plays a crucial role in various pathophysiological processes and diseases, such as tumours, myocardial infarction, heart failure, myopia, Alzheimer's disease, autophagy, ferroptosis, etc. Therefore, in‐depth research on lactylation can expand our understanding of cell metabolism and the basic principles of biology.

Kla includes L‐Kla and D‐Kla, among them L‐Kla being the most predominant. Therefore, unless otherwise specified, Kla in this article refers to L‐Kla.

## 2 Advances of Lactylation

This section will sequentially introduce the discovery, molecular mechanisms, stereoisomers, regulatory factors, modification sites and detection methods of lactylation.

### 2.1 Discovery

In 2019, Zhang et al. [8] discovered that three proteolytic enzymes exhibited a mass shift of 72 Da on certain lysine residues, which is consistent with the shift caused by lactylation. To validate it, Zhang D compared artificially synthesised lactylated peptides with the identified modified peptides, and found them shifted in the same manner. Based on these, Zhang D formally proposed the concept of lactylation.

### 2.2 Stereoisomers

There are three stereoisomers for Kla: L‐lactylation (L‐Kla), D‐lactylation (D‐Kla) and N‐ε‐carboxyethyllysine (Kce). Among them, L‐Kla is the most predominant, accounting for over 90% [9]. Therefore, unless otherwise specified, Kla in this article refers to L‐Kla.

L‐Kla, D‐Kla and Kce have the same molecular weight and similar structures, making it difficult to distinguish. Fortunately, specific monoclonal antibodies for each isomer have been developed. In this way, Western blotting can distinguish them, but mass spectrometry (MS) still cannot. Finally, through a chiral derivatisation reaction, by introducing additional chiral chemical groups to expand the stereochemical differences, MS can also be used for differentiation.

In addition, glucose metabolism only regulates L‐Kla, while hardly affecting D‐Kla or Kce. Inhibitors of enolase (ENO) can reduce L‐Kla and increase D‐Kla, while having little effect on Kce. Inhibitors of LDHA only reduce L‐Kla, without affecting D‐Kla or Kce. The glyoxalase pathway only regulates D‐Kla and Kce, and does not affect L‐Kla.

### 2.3 Molecular Mechanism

**FIGURE 1:**
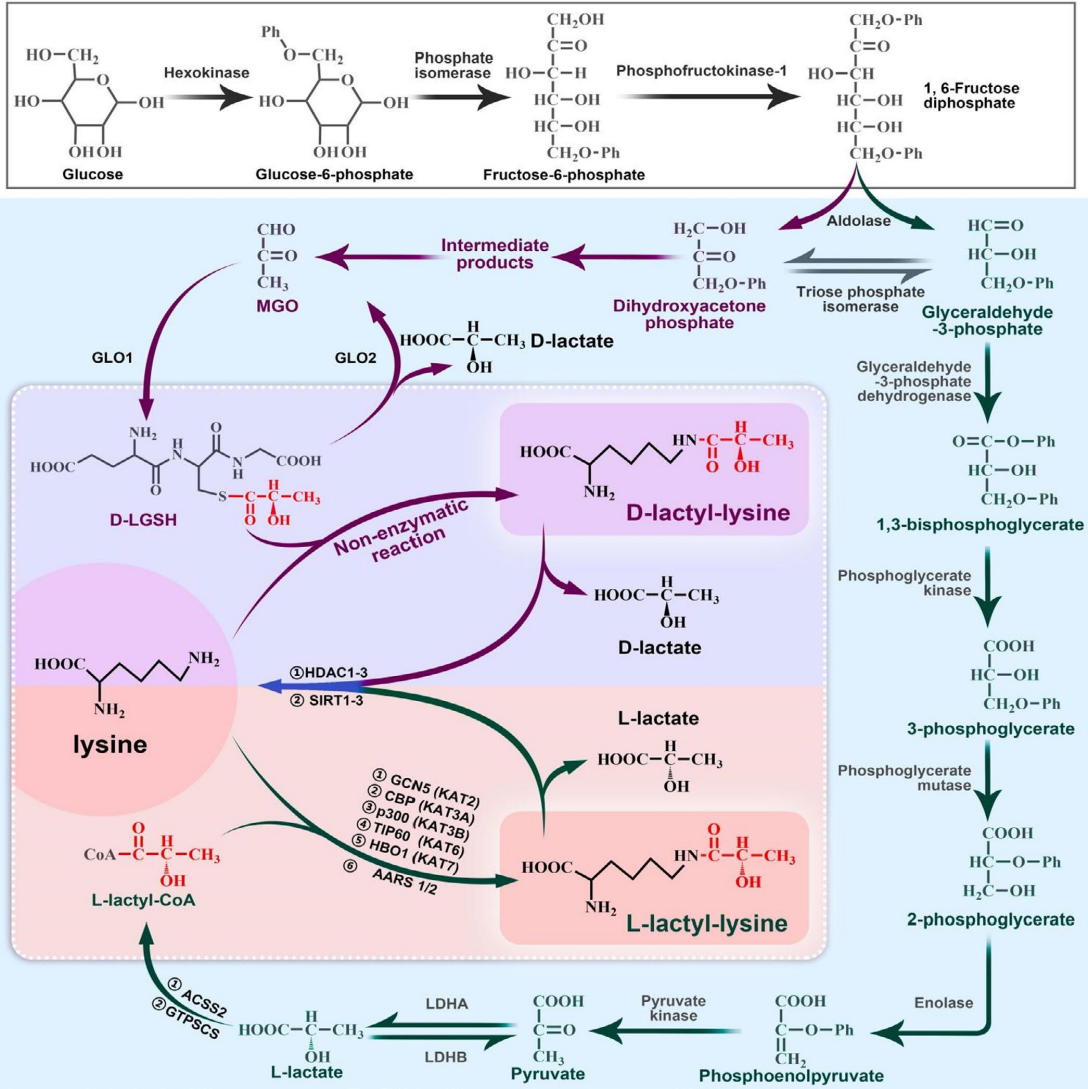
Mechanism of lactylation. Two mechanisms of lactylation: L‐lactyl‐CoA pathway and D‐lactoylglutathione pathway. Although taking different paths, both are side routes of the glycolytic pathway. In the thoughts of Zhang D, L‐lactyl‐CoA is the direct substrate and the ‘writer’ is P300/CBP. But recent research has identified more ‘writers’, including GCN5 (KAT2), TIP60 (KAT6), HBO1(KAT7), KAT8 and AARS. In the thoughts of Gaffney, the LGSH is the direct substrate and the reaction is non‐enzymatic. The ‘eraser’ for both pathways are HDAC1‐3 and SIRT1‐3.

Understanding the mechanism of lactylation is crucial for comprehending its regulation. Two teams have explored but get different conclusions. The discrepancy may stem from the differing focuses, one on L‐Kla of histones while the other on D‐Kla of non‐histones (Figure 1).

#### 2.3.1 L‐Lactyl‐CoA Pathway for L‐Lactylation

The overall reaction is: L‐lactyl‐CoA + Lysine → L‐lactyl‐lysine + CoA.

Zhang et al. [8] thought that L‐Kla is a post‐translational modification that uses L‐lactyl‐CoA as substrate and depends on the ‘writer’ p300, primarily affecting gene transcription.

Varner et al. [10] validated the presence of L‐lactyl‐CoA and measured its concentration in mouse cardiac tissue at 1.14 pmol/L.

#### 2.3.2 D‐Lactoylglutathione Pathway for D‐Lactylation

The overall reaction is: D‐LGSH + Lysine → D‐lactyl‐lysine + GSH.

Gaffney et al. [11] believes that D‐lactoylglutathione (D‐LGSH), generated by the glycolytic by‐product methylglyoxal (MGO) with glutathione (GSH) under the catalysis of glyoxalase 1 (GLO1), is the direct substrate for D‐Kla. This is a passive, non‐enzymatic reaction driven by pH. It can be supported by the fact that stable H4‐Kla occurs when H4 and D‐LGSH are coincubated in vitro.

### 2.4 Regulatory Factors

Multiple factors are involved in the regulation of lactylation, including lactate concentration, lactate transporting enzymes, ‘writers’ ‘erasers’ and crosstalks.

#### 2.4.1 Lactate Concentration

L‐lactate and D‐lactate have different sources, metabolic enzymes, physiological functions and disease significance (Table 1).

**TABLE 1:** L‐lactate and D‐lactate.

	L‐lactate	D‐lactate
Chemical configuration	L‐S configuration (levorotatory)	D‐R configuration (dextrorotatory)
Biological activity	Yes	No
Source	Glycolysis	1. MGO pathway 2. gut microbes
Blood concentration	1–2 mmol/L	10 μmol/L
Metabolic enzymes	LDH	D‐2‐hydroxyacid dehydrogenase
Physiological functions	1. L‐lactylation 2. energy supply 3. pH regulation	Intestinal immune microenvironment
Clinical significance	Marker of tissue hypoxia	Marker of intestinal barrier
Associated diseases	Tumours Hypoxia Shock Infections	Diabetes Short‐bowel syndrome Intestinal ischemia

L‐lactate promotes L‐Kla in a dose‐dependent manner. The concentration of L‐lactate in normal human blood is around 1 mmol/L, but it can exceed 15 mmol/L under conditions such as exercise, infection and shock [12]. Additionally, sodium lactate and mitochondrial inhibitors (such as rotenone) can enhance L‐Kla, while glycolysis inhibitors (such as sodium dichloroacetate [DCA] and sodium oxalate [OXA]) and glucose analogs (such as 2‐deoxy‐D‐glucose [2‐DG]) can reduce it.

D‐lactate mainly comes from the MGO pathway and gut microbes, and its content is extremely low, only about 10 μmol/L. D‐lactate is a biomarker of the intestinal barrier. In cases of gut microbiota dysbiosis such as short‐bowel syndrome, D‐lactate can exceed 3 mmol/L [13, 14]. D‐LGSH is the substrate for D‐Kla, which can be hydrolysed by GLO2 into D‐lactate. In the state of immune activation, GLO2 is inhibited by the NF‐κB signalling pathway, leading to the accumulation of D‐LGSH and thereby enhancing D‐Kla [15].

In addition, oxygen concentration is also directly related. In theory, under hypoxic conditions, glycolysis is enhanced and leads to accumulated lactate. But Yang et al. [16] pointed out that under low oxygen conditions (< 2%), the lactylation levels in mouse embryos are reduced. However, under low oxygen conditions, the metabolic and DNA expression themselves decrease, so it cannot be directly concluded that low oxygen is correlated with decreased lactylation. To eliminate the potential influence of low oxygen on cellular metabolism, Hou et al. [17] conducted a study on mice adapted to high‐altitude hypoxia and found increased lactylation. This is consistent with the theoretical notion that glycolysis and lactylation are enhanced under low oxygen concentration.

#### 2.4.2 Lactate Transporting Enzymes

Lactate molecule is polar and hydrophilic, making it difficult to directly pass through the cell membrane. Monocarboxylate transporters (MCTs) are protein channels located in the cell membrane responsible for the transport of monocarboxylic acids, such as lactate and pyruvate. MCTs include various subtypes. In specific cells or tissues, the transport direction of these subtypes differs. MCT1 primarily mediates inward transport, while MCT4 primarily facilitates outward transport and is related to lactic acidosis environments (Table 2).

**TABLE 2:** MCT subtypes.

	MCT1	MCT2	MCT4
Location	Red muscle cell Erythroid cell	Neuron Astrocyte	White muscle cell Tumour tissue
Substrate	Lactate, pyruvate	Mainly lactate	Mainly lactate
Direction	Inward	Usually inward, Can also outward	Inward
Inhibitor	Specific: AZD3965		
	Specific: AR‐155858		
	Non‐specific: α‐cyano‐4‐hydroxycinnamate (4‐CIN)		

#### 2.4.3 Regulating Enzymes

Lactyl regulating enzymes including ‘writers’ and ‘erasers’. Enzymes have a relative specificity for substrate affinity, thus some acetyl regulating enzymes can also regulate lactylation. Moreover, lactate level is significantly higher than lactyl‐CoA; the novel lactylation regulatory model based on lactate rather than lactyl‐CoA further expands the substrate range for lactylation.

The discovered ‘writers’ and ‘erasers’ are listed. (Table 3).

**TABLE 3:** Writers and erasers.

	Types	Targets	Associated diseases	Agonists		Inhibitors	
				Specific	Non‐specific	Specific	Non‐specific
Writers	CBP (KAT3A)/p300 (KAT3B)	Most significant and widespread [8]		—	—	A485 C646	—
	GCN5 (KAT2A)	H3K14, K18	Tumours [18]	—		CPTH2 MB‐3	
		H3K18	Myocardial infarction [19]				
	TIP60 (KAT6)	NBS1‐K388	Gastric cancer [20]	—		TH1834	
	HBO1 (KAT7)	H3K9	Cervical cancer [21]	—		WM‐3835	
	MOF (KAT8)	eEF1A2‐K408	Colorectal cancer [22]	—		CHI‐KAT8i5	
		PCK2‐K100	Liver ischemia–reperfusion injury [23]				
	AARS1	p53‐K120, K139	Breast cancer [24]	—	—	—	β‐Alanine
		YAP‐K90 TEAD1‐K108	Gastric cancer [25]				
		IF116‐K90	Virus infection [26]				
	AARS2	cGAS‐K131, K156	Innate immunity [27]	—		—	
Erasers	HDAC1	Most significant and widespread		—	—	MGCD0103	MS‐275 vorinostat trichostatin A
	HDAC2	H3K9	Pathological neovascularisation [28]	—		Santacruzamate A	
		H3K18	Pancreatic ductal adenocarcinoma [29]				
	HDAC3	H4K12	Atherosclerosis [30]	—		BG45	
	SIRT1	α‐MHC‐K1897	Heart failure [31]	SRT2104	Nicotinamide‐ Riboside Nicotinamide‐ Mononucleotide	EX‐527	SIRT‐IN‐1 Nicotinamide
		YAP‐K90 TEAD1‐K108	Gastric cancer [25]				
		MRE11‐K673	Breast cancer [32]				
	SIRT2			S‐KET		Tubastatin A	
	SIRT3	ACSL4‐K412	Intervertebral disc degeneration [33]	TND1128		YC8‐02	
		ALDH2‐K52	Acute kidney injury [34]				

##### 2.4.3.1 Writers

Lysine acetyltransferases (KATs) can bind to various acyl‐CoA to transfer acetyl, lactyl [8], propionyl, butyryl groups [35]. Traditionally, CBP (KAT3A) and p300 (KAT3B) were considered the primary lactyltransferase, and p300 can also exert a synergistic effect with p53 in a p53‐dependent and p300‐mediated manner [36]. However, recent studies have shown that GCN5 (KAT2A), TIP60 (KAT6), HBO1 (KAT7), MOF (KAT8) and alany‐tRNA synthetase (AARS) also possess lactyltransferase activity, which will be discussed in detail in ‘Disease Associations’ section.

It is worth mentioning that the function of the ‘writer’ can be blocked by mutations from lysine (K) to arginine (R).

##### 2.4.3.2 Erasers

Delactylases mainly include histone deacetylases (HDAC1‐3) and sirtuins (SIRT1‐3).

In terms of delactylation intensity, HDAC1‐3 are the most important, which are 1000 times stronger than SIRT1‐3 [37]. Among the SIRTs, SIRT1 and SIRT3 are stronger than SIRT2 [38].

Regarding substrate preference, HDAC1‐2 prefer histones, HDAC3 has a strong effect on both histones and non‐histones, and SIRTs prefer non‐histones. It is worth mentioning that SIRT1 preferentially recognises glycine‐rich sequences and can regulate glycolytic enzymes lactylation, such as pyruvate kinase PKM‐K207la [38]. SIRT3 is a specific ‘eraser’ for mitochondrial lactylated proteins. Inhibiting SIRT3 leads to an abnormal increase in mitochondrial lactylation, which in turn causes ROS burst and cell apoptosis [34].

Regarding lactylation types, both HDACs and SIRTs show a significant preference for L‐Kla, only HDAC3 having a strong delactylation effect on D‐Kla [15].

##### 2.4.3.3 Lactyl‐CoA Synthetase

As of May 2025, two lactyl‐CoA synthetases have been identified: acetyl‐CoA synthetase 2 (ACSS2) and GTP‐dependent succinyl‐CoA synthetase (GTPSCS).

Given that epidermal growth factor receptor (EGFR) promotes the Warburg effect to increase lactate, Zhu R.X found H3K14la and H3K18la were the most upregulated, and the ‘writer’ was KAT2A. Mechanistically, EGF mediated ACSS2 nuclear translocation and ultimately formed LDHA/ACSS2/KAT2A complex that serves as a lactyltransferase [18].

Liu RL [39] knocked down several acyl‐CoA synthetase genes and found that knockdown of GTPSCS significantly reduced lactylation. To clarify the molecular mechanism by which GTPSCS, as a mitochondrial enzyme, acts as a lactyl‐CoA synthetase to catalyse histone lactylation, the authors revealed the nuclear localisation signal sequence: 192‐KKGR‐195. Mutating K192 inhibited GTPSCS nuclear translocation and significantly reduced lactyl‐CoA and histone lactylation levels. Additionally, the downstream ‘writer’ was p300, with the primary target being H3K18la. In summary, in the nucleus, the GTPSCS/p300 complex can directly generate lactyl‐CoA, and p300 can directly use lactyl‐CoA to catalyse H3K18la. Moreover, the highly efficient acyltransferase activity of p300 further promotes the synthesis of lactyl‐CoA by GTPSCS.

#### 2.4.4 Crosstalks

Protein modification networks constitute a sophisticated system where various chemical modifications, such as acetylation, lactylation, succinylation, methylation and ubiquitination, can dynamically interact through crosstalks.

Taking acetylation as an example, it preferentially targets lysine residues, too. The concentration of acetyl groups is far greater than lactyl groups, and the concentration of lactyl‐CoA is only 1/350 to 1/20 of acetyl‐CoA [10]. Acyltransferases have relative specificity and can recognise both acetyl and lactyl groups. These factors lead to acetylation binding to lysine residues through competitive inhibition, effectively ‘occupying’ the sites for lactylation. Latham [40] found that lactate can inhibit the activity of deacetylases, thereby enhancing acetylation. Zhang et al. [8] further discovered that acetylation (Kac) and lactylation (Kla) have different temporal dynamics during macrophage polarisation. Kac peaks at 6 h while Kla peaks at 24 h, indicating slower kinetics for Kla. Additionally, 68% (1223/1787) of detected histone sites were H3K18la. This indicates a strong enrichment of Kla. Furthermore, Zhang D subjected HeLa cells and mouse macrophages to hypoxia treatment and found decreased Kac and increased Kla in HeLa cells, while increased Kla and almost unchanged Kac in mouse macrophages. Therefore, it can be concluded that Kac and Kla exhibit species‐specific and cell‐specific characteristics.

### 2.5 Modification Sites

Lactylation sites can be primarily categorised into histone sites and non‐histone sites. Some significant sites, along with their research findings, will be integrated and displayed in ‘Disease Association’ section (Table 4).

**TABLE 4:** Associations between lactylation and diseases.

Site		Models	Result	Ref
Pan‐Kla	HMGB1	Acute kidney injury	HMGB1‐Kla drives neutrophil extracellular trap formation in lactate‐induced acute kidney injury	[41]
		Pulmonary fibrosis	TGF‐β1 induces pulmonary fibrosis through Kla induced fibrosis‐related genes	[42]
	HMGB1	Sepsis	HMGB1‐Kla promotes the translocation from nucleus to cytoplasm and leads to the deterioration of sepsis	[43]
D‐Kla	RelA‐K310	Immune response	D‐LGSH mediates RelA‐D‐K310la through NF‐κB pathway thereby negatively inhibiting the immune response	[15]
Histone sites	H3K9	Pathological neovascularisation	‘VEGF‐HDAC2‐H3K9la’ axis promotes angiogenesis	[28]
		Cervical cancer	HBO1 is the ‘writer’ of H3K9la which activates the expression of tumour‐associated genes	[21]
	H3K14 H3K18	Tumours	ACSS2 acts as lactyl‐CoA synthetase and couples KAT2A for histone lactylation and tumour immune evasion	[18]
	H3K18	Tumours	H3K18la promotes the polarisation from M1‐inflammatory type to M2‐repareative type	[8]
		Cell senescence	Glis1 closes somatic genes and opens glycolytic genes via ‘epigenome‐metabolome‐epigenome’ signalling cascade	[44]
		Glioma	GTPSCS functions as a lactyl‐CoA synthetase to promote histone lactylation and gliomagenesis	[39]
		Embryonic development	H3K18la regulates major zygotic genome activation in mammal embryonic development	[45]
		Myopia	‘Glycolysis—H3K18la—Notch1’ axis induces myopia	[46]
		Myocardial infraction	H3K18la can enhance the repairing capability of macrophage and improve the prognosis of myocardial infarction	[19]
		Pancreatic ductal adenocarcinoma	Positive feedback of ‘LDHA‐H3K18la‐TTK/BUB1B‐LDHA’ exacerbates tumour progress	[29]
		Septic shock	H3K18la may serve as a diagnostic and monitoring marker in septic shock	[47]
	H3K9 H3K56	Liver cancer	DML inhibits liver cancer stem cell by inducing apoptosis through Bax/Bcl2/Caspase‐8 signalling pathway	[48]
	H3K18 H4K8 H4K12	Alzheimer's Disease	‘IDH3β‐lactate‐*Pax6*‐IDH3β’ positive feedback inhibits the expression of IDH3β thus deteriorates AD	[49]
	H4K12	Alzheimer's Disease	‘PKM‐H4K12la‐*Pkm*’ positive feedback promotes the expression of *Pkm* thus deteriorates AD	[50]
		Atherosclerosis	‘TRAP1‐HDAC3‐H4K12la’ axis promotes senescence‐associated secretory phenotype genes	[30]
		Kidney fibrosis	‘PFKFB3‐H4K12la‐NF‐κB’ axis promotes inflammatory response and leads to kidney fibrosis	[51]
Histone and non‐histone	H3K56 ALDOA‐K230/322	Liver cancer	ALDOA‐K230/322la and H3K56la jointly induce the stemness remodelling of liver cancer stem cells	[52]
Non‐histone sites	ALDH2‐K52	Acute kidney injury	ALDH2‐K52la exacerbates renal injury by disrupting PHB2‐mediated mitophagy	[34]
	PCK2‐K100	Ischemia–reperfusion	KAT8 induces PCK2‐K100la to exacerbates hepatic ferroptosis in ischemia/reperfusion injury	[23]
	ACL4‐K412	Intervertebral disc degeneration	ACL4‐K412la activates ferroptosis during intervertebral disc degeneration	[33]
	cGAS‐K156	Infections	AARS2 mediated cGAS‐K156la induces immunosuppression	[24]
	p53‐K120, K139	Breast cancer	AARS1 mediated p53‐K120la and K139la is essential for tumorigenesis	[27]
	IFI16‐K90 RBM14‐K600	Virus infections	IFI16‐K90la and RBM14‐K600la inhibit DNA damage response and virus infections immune	[26]
	SNAP91‐K885	Stress	Exercise can enhance synaptic structure formation to relieve stress by increasing SNAP91‐K885la	[53]
	ARF1‐K73	Ischemic stroke	Inhibiting the ‘LRP1‐ARF1‐K73la’ axis can promote mitochondrial transport and improve stroke prognosis	[54]
	YY1‐K183	Retinopathy	Inhibiting the ‘glycolysis‐YY1‐K183la‐‐FGF2’ axis can reduce angiogenesis thereby relieve retinopathy	[55]
	lkzf1‐K164	Autoimmune uveitis	lkzf1‐K164la mediated abnormal differentiation of Th17 cell is essential for autoimmune uveitis	[56]
	α‐MHC‐K1897	Heart failure	Reduced α‐MHC‐K1897la can deteriorate heart failure while MCT4 inhibitor can relieve it	[31]
	MRE11‐K673	Breast cancer	MRE11‐K673la enhances homologous recombination repair and induce tumour chemotherapy resistance	[32]
	NBS1‐K388	Gastric cancer	NBS1‐K388la promotes formation of ‘MRE11‐RAD50‐NBS1’ complex thus leading to chemotherapy resistance	[20]
	YAP‐K90 TEAD1‐K108	Gastric cancer	AARS1 can catalyse YAP‐K90la and TEAD1‐K108la thereby exacerbating gastric cancer	[25]
	eEF1A2‐K408	Colorectal cancer	KAT8 is the ‘writer’ of eEF1A2‐K408la which promotes tumour gene expression	[22]
	XRCC1‐K247	Glioblastoma	XRCC1‐K247la promotes its nuclear localisation and enhances DNA repair thus leading to resistance	[57]
	AK2‐K28la	Tumours	AK2‐K28la inhibits its phosphokinase activity and leads to energy metabolism disorders and tumour cell proliferation	[58]
	SORBS3‐K479	Liver fibrosis	SORBS3‐K479la activates hepatic stellate cells and exacerbates liver fibrosis	[59]

#### 2.5.1 Histone Sites

Histones and DNA are closely connected to form chromosomes. DNA is relatively rigid, so it faces great challenges in replication and transcription without auxiliary factors. This is where histones come into effect. The sequences of histones are highly conserved throughout evolution, with H2A, H2B, H3 and H4 forming the nucleosome core. Histones undergo various PTMs, which can alter the structure and accessibility of chromatin, making it either relaxed (facilitating gene expression) or condensed (leading to gene silencing), thereby exerting a gene regulatory effect [60].

**FIGURE 2:**
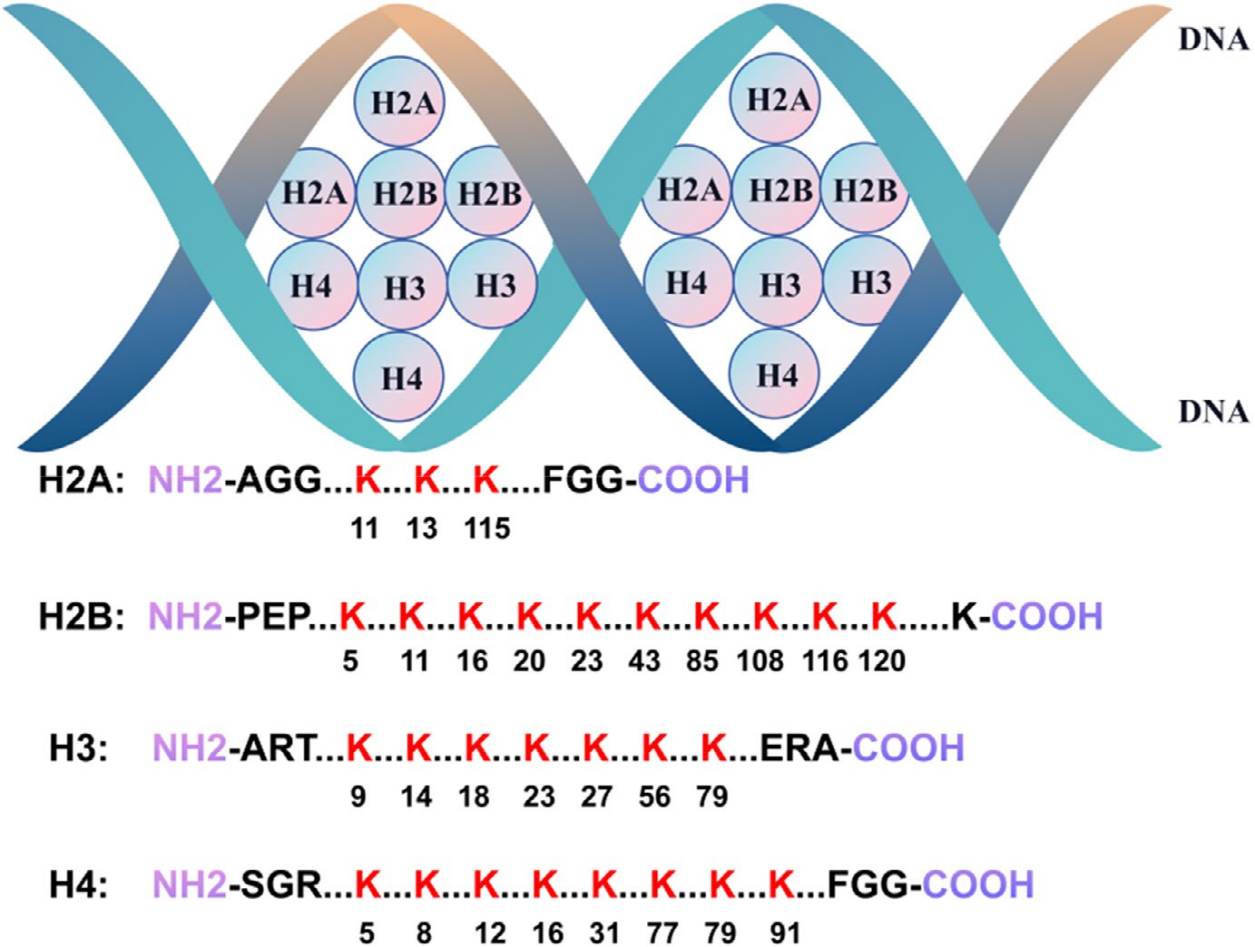
Histone lactylation sites. Histones and DNA are tightly bounded. The histone core is an octamer composed of H2A, H2B, H3 and H4. All Kla sites of the histones are shown according to the amino acid sequence of the peptide chai.

Identified histone lactylation sites are demonstrated (Figure 2), including H3K9, H3K14, H3K18, H3K27, H4K8, H4K12.

Zhang et al. [8] first proposed lactylation and found it predominantly occurs at H3K18, with enrichment at the promoters of homeostatic genes such as arginase 1 (*Arg1*). H3K18la facilitates the polarisation of macrophages from the pro‐inflammatory M1 type to the anti‐inflammatory M2 type.

Pan et al. [48] discovered that demethylzeylasteral (DML) inhibits tumour progression by suppressing H3K9la in tumour stem cells.

Pan et al. [50] observed a significant increase of H4K12la in microglia in Alzheimer's disease. H4K12la enriched in promoters of glycolytic genes, especially *Pkm*, and formed a positive feedback of ‘glycolysis‐*Pkm*‐H4K12la‐*Pkm*‐glycolysis’.

#### 2.5.2 Non‐Histone Sites

With the advancement of research, a large number of non‐histone lactylation sites in the cytoplasm, mitochondria and nucleus have been identified. In terms of molecular weight, lactylated proteins are mostly around 25 kDa [17]; in terms of protein structure, lactylation predominantly occurs in α‐helical regions [61]. Some special non‐histone lactylation sites are listed (Table 4).

Zhang et al. [62] identified 257 lactylated proteins and 387 lactylation sites on the protozoan parasite. In terms of site quantity, 76% (195/257) had 1 lactylation site, 14% (35/257) had 2 sites and 5% (14/257) had three sites. In terms of sub‐cellular localisation, the nucleus accounts for the largest proportion, 38% (98/257), followed by the cytoplasm at 35% (90/257) and mitochondria at 11% (29/257). This suggests that lactylation plays a more widespread role in transcription and translation.

Gaffney et al. [11] identified 350 lactylated proteins in HEK293T cells and found them enriched in carbohydrate metabolism, such as aldolases (ALDO), phosphoglycerate kinase, pyruvate kinase (PKM) and glyoxalase 1 (GLO1). These lactylated enzymes can regulate glycolysis through negative feedback.

### 2.6 Detection Methods

Detection of lactylation is a prerequisite. Existing techniques include isotope labeling, pan‐kla or site‐specific antibodies, MS‐based proteomics and bioorthogonal detection.

In the isotope labeling method, lactate is labelled with ^13^C or ^2^H, and then the modification sites can be dynamically and accurately detected. However, the method is cumbersome, expensive and the radioactivity poses certain risks. Moreover, it cannot be used for protein content enrichment.

Pan‐Kla antibody can broadly recognise all Kla sites. It can quantitatively compare the total levels in WB and can clarify the localisation in tissues or sub‐cellular structures in immunostaining. Pan‐Kla antibody is cheap and easy to use, but it lacks specificity.

Site‐specific antibodies can precisely explore the functions and regulatory factors of the target proteins Kla sites, especially histone Kla antibodies can be combined with CUT‐Tag to analyse the regulatory relationship between histone Kla and target genes. However, their development is difficult and time‐consuming, and they have poor applicability across different species.

MS‐based proteomics has high‐throughput analysis capabilities and can systematically conduct analyses such as functional pathway enrichment and protein–protein interaction networks. However, during the peptide digestion process, information loss is inevitable, so its sensitivity needs to be improved. In addition, Gao et al. [63] developed Customized Histone Mark Analysis (CHiMA), which uses deep‐learning computer‐assisted spectral analysis to improve the traditional MS analysis process, significantly enhancing the detection ability for low‐abundance or unknown histone modifications. It newly identified 26 histone Kla sites, such as H1K33, H2AK36, H2BK34, H3K56 and H4K79.

Bioorthogonal chemistry, also known as live‐cell chemical modification, refers to the modification or introduction of new biological components without generating toxic substances and minimally interfering with cellular activities. Based on this, Sun et al. [64] proposed a novel bioorthogonal lactyl analog, sodium‐(S)‐2‐hydroxypent‐4‐ynoate (YnLac). YnLac directly targets lysine residues, allowing for more efficient detection of Kla and identification of modification sites.

## 3 Relationships Between Lactylation and Cellular Functions

Lactylation connects metabolism and epigenetics regulation, playing an important role in macrophage polarisation, immune and inflammatory responses, ferroptosis, cell fate determination.

### 3.1 D‐Lactate and D‐Kla in Immune and Inflammatory Responses

As mentioned above, D‐lactate not only comes from the MGO pathway but also from gut microbes, which can be absorbed into the blood via the portal vein. Currently, there are few studies on D‐Kla, which are limited to immune and inflammatory responses.

Macrophages are an important part of the immunosuppressive microenvironment and often exhibit M2 polarisation. Han et al. [65] found that D‐lactate promoted macrophage polarisation from M2 to M1, where M2 markers (ARG1, CD206, IL‐10) were downregulated and M1 markers (TNF‐α, iNOS, IL‐12) were upregulated. This means that D‐lactate can reverse the immunosuppressive microenvironment and restore the killing function of M1 macrophages. Mechanistically, D‐lactate binds to the TLR2/TLR9 receptors on the surface of macrophages, thereby inhibiting PI3K/Akt but activating the NF–κB pathway. In addition, a D‐lactate targeted delivery system, namely poly(lactic‐co‐glycolic acid) (PLGA) nanoparticles and M2 macrophage‐binding peptide (M2pep) was developed. It can also combine with anti CD47 antibody to activate CD8^+^ T cells and NK cells to exert multiple effects, ultimately reducing the tumour volume by more than 80%.

The immune system has a delicate regulation, where moderate immune response is generally beneficial for resisting infections while abnormal activation often leads to damage such as autoimmunity and cytokine storms. Zhao et al. [15] found that the activation of the NF‐κB pathway in macrophages decreased glyoxalase GLO2, and GLO2 was associated with abnormally activated immune responses. Mechanistically, NF‐κB induces Tristetraprolin (TPP) to degrade the mRNA of *Glo2*, thus weakening hydrolysing D‐LGSH to generate D‐lactate and leading to a significant occurrence of D‐Kla. Lactylation omics showed the differentially modified proteins were enriched in immune and inflammatory pathways, with RelA‐D‐K310la being the most prominent. D‐Kla inhibits the nuclear translocation of RelA, thereby suppressing the expression of NF‐κB mediated pro‐inflammatory factors such as TNF and IL‐6, and ultimately inhibiting the activation of immune response. Knockout of *Glo2* improved the survival rate of the inflammatory mouse model, while overexpression of *Glo2* exacerbated the immune response. This study reveals that D‐LGSH mediates D‐Kla through the NF‐κB pathway, thereby negatively inhibiting the immune response, providing new insights for diseases such as excessive inflammation and autoimmunity.

### 3.2 L‐Lactate and L‐Lactylation

#### 3.2.1 Macrophage Polarisation

Macrophages can polarise into M1 and M2 states, each with distinct functions. M1 type represents the classical activation state, which is activated by IFN‐γ, TNF‐α or LPS, and releases pro‐inflammatory mediators such as IL‐1, IL‐6 and TNF. After metabolic reprogramming, M1 macrophages favour aerobic glycolysis and lead to accumulated lactate. In contrast, M2 type represents an atypical activation state, activated by IL‐4 or IL‐13, and secretes anti‐inflammatory mediators such as IL‐10, TGF‐β. After metabolic reprogramming, M2 macrophages favour OXPHOS and lead to decreased lactate.

**FIGURE 3:**
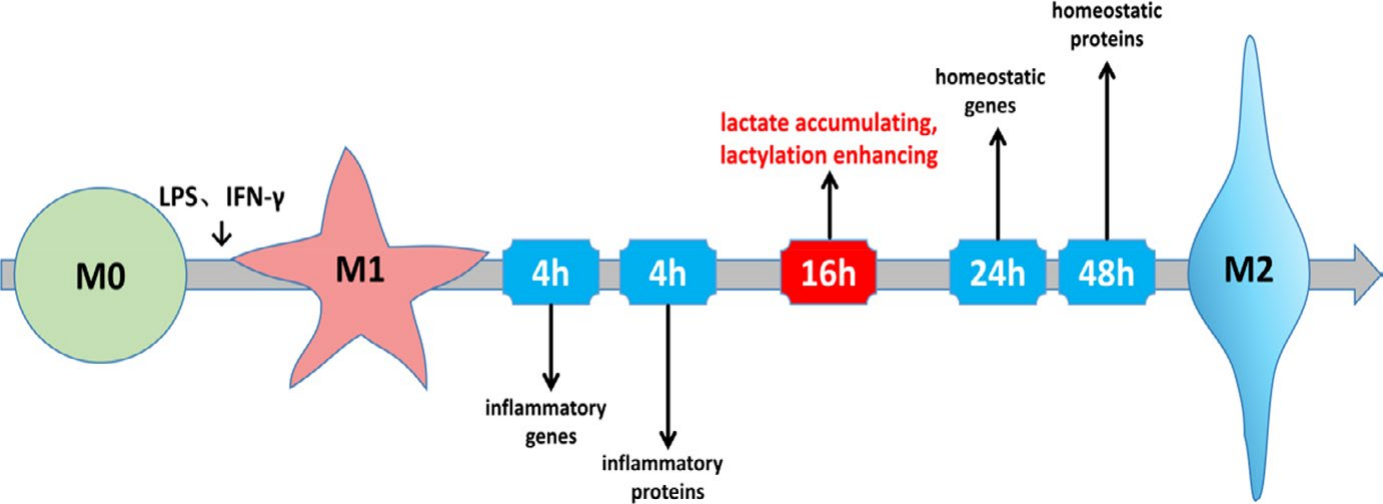
Lactate clock. During the process of macrophage M1 polarisation, there are sequential occurrences of inflammatory gene expression, inflammatory proteins peak, enhanced lactylation, homeostatic gene expression and homeostatic proteins peak. Lactylation acts like a clock, initiating the transition from the M1 phenotype to the M2 phenotype.

Zhang et al. [8] stimulated BMDMs with LPS and IFN‐γ to induce M1 polarisation. It was found that pro‐inflammation genes such as *iNos* and *Il‐6* were significantly expressed within 4 h, lactate and lactylation significantly increased after 16 to 24 h, and the expression of homeostatic genes such as arginase 1 (*Arg1*) was detected after 48 h. Arginine metabolism is regulated during macrophage polarisation. M1 macrophages have low levels of ARG1 and use iNOS to produce NO from arginine to kill pathogens. In contrast, M2 macrophages have high levels of ARG1 and utilise arginine to produce ornithine, promoting damage healing. These suggest that after macrophage activation, the gradually accumulated lactate and increased lactylation lead to a transition from M1 to M2, enabling the expression of homeostatic genes and facilitating the repair of inflammatory damage (Figure 3).

Regarding this phenomenon, Li L [44] proposed that the transcription factor GLIS family zinc finger 1 (Glis1) plays a crucial role. Glis1 itself can reprogram senescent cells into pluripotent cells to enhance genomic stability. In the early stages of reprogramming, Glis1 directly binds to chromatin to assist in the silencing of somatic genes and opening of glycolytic genes such as enolase 1 (*Eno1*) and aldolase, markedly enhancing glycolysis and lactylation. Elevated H3K18la further assists in the reprogramming of senescent cells, activating genes that induce pluripotent stem cells, such as *Oct4*, *Sall4* and *Mycn*. Thus, a signal cascade of ‘epigenome‐metabolome‐epigenome’ is formed. Therefore, during the early stages of M1 polarisation, histone lactylation may be temporarily delayed until the cascade reaction of Glis1 takes effect (approximately 16‐24 h), which leads to a significant increase in lactylation and promotes M2 polarisation.

In summary, activated macrophages have an endogenous ‘lactate clock’ that can spontaneously promote the transition from M1 to M2 polarisation.

#### 3.2.2 Cell Stress and Death

Ferroptosis is an iron‐dependent cell death driven by excessive lipid peroxides. In essence, it is an ROS burst caused by the depletion of glutathione and glutathione peroxidase [66].

Yuan et al. [23] found that lactate exacerbates liver ischemia–reperfusion injury through ferroptosis, while the ferroptosis inhibitor can alleviate it. Lactylation omics identified phosphoenolpyruvate carboxykinase PCK2‐K100la as a key target. PCK2‐K100la enhances its interaction with 3‐oxoacyl‐ACP synthase (OXSM), thereby stabilising its protein level and ultimately activating the ferroptosis pathway. Overexpression of PCK2 can exacerbate liver ischemia–reperfusion injury and the ferroptosis phenotype. In addition, its ‘writer’ is KAT8. Inhibiting the ‘KAT8‐PCK2‐OXSM’ axis can mitigate liver ischemia–reperfusion injury and ferroptosis mediated by hyperlactatemia.

Sun et al. [33] found that in nucleus pulposus cells during intervertebral disc degeneration, glycolysis was enhanced while OXPHOS was weakened. In addition, lactate mediates H3K18la to promote the expression of long‐chain acyl‐CoA synthetase 4 (ACSL4), a key molecule related to ferroptosis. This phenomenon can be blocked by the p300/CBP inhibitor A485. Moreover, ACSL4‐K412laenhances its dimerisation and enzymatic activity, thus aggravating ferroptosis. It is worth mentioning that SIRT3 is the ‘eraser’ of ACSL4‐K412la. In conclusion, lactate simultaneously mediates the dual regulation of H3K18la and ACSL4‐K412la to activate ferroptosis.

#### 3.2.3 Immune Regulation

##### 3.2.3.1 Tumour Immunity

p53 is a critical tumour suppressor. In breast cancer, Zong et al. [24] found that p53 K120/K139la leads to its inactivation and leads to tumour progression, while *Ldha* knockout reduced tumour burden and increased p53 activity. Furthermore, CRISPR screen revealed the strongest association with alany‐tRNA synthetase 1 (AARS1). Knockdown of AARS1 decreased 80% Kla, while its overexpression significantly increased Kla, indicating that AARS1 is a key ‘writer’. Mechanistically, lactate in AARS1 is activated by ATP to form an activated intermediate lactate‐AMP compound, which covalently binds to lysine, releasing AMP and completing lactylation. Additionally, β‐alanine is an inhibitor of AARS1‐mediated lactylation by competing binding. In summary, AARS1 acts as both a lactate sensor and lactyltransferase to mediate tumorigenesis through p53‐K120/K139la, and β‐alanine is a new target to enhance chemotherapy efficacy.

##### 3.2.3.2 Innate Immunity

Cyclic GMP‐AMP synthase (cGAS) is a cytosolic DNA sensor responsible for combating microbial invasion by stimulating immune responses through ‘cGAS‐STING’ signalling pathway. Li et al. [27] found cGAS‐K131la in humans and cGAS‐K156la in mice inhibited diminished its liquid–liquid phase separation ability, thus inhibiting the cGAS‐mediated immune respons. Additionally, the cGAS‐K156Q point mutation mouse model infected with HSV exhibited more severe injury and increased mortality, suggesting suppressed immune function. Mechanistically, cGAS can recognise foreign DNA, while lactylated cGAS cannot sense. Furthermore, the ‘writer’ was AARS2. MCT1 or AARS2 inhibitors can block lactate uptake or cGAS lactylation, thus preventing immunosuppression.

##### 3.2.3.3 Virus Infection Immunity

Matthew's et al. [26] study on HCMV infection showed that the DNA sensor IFI16‐K90la blocks the nuclear recruitment of the DNA damage kinase DNA‐PK, thereby inhibiting the DNA damage response and IFN signalling pathway. The RNA‐binding protein RBM14‐K600la suppressed the antiviral RNA metabolism pathway, thus promoting replication of the virus. In addition, in HSV‐1 infection, IFI16‐K90la also exists, suggesting a common mechanism of lactylation in inducing immune evasion during herpesvirus infections, such as HCMV and HSV‐1. Moreover, AARS1 is the most prominent ‘writer’. OXA, AARS1 knockdown or alanine can restore antiviral ability.

#### 3.2.4 Cell Fate Decision

During early embryonic development, glycolysis dominates due to relative hypoxia and ischaemia [67], which provides the necessary conditions for lactylation to occur.

Tissue‐specific gene regulatory networks (GRNs) govern embryonic cell differentiation, determining cellular identity and function. In neural crest cells (NCCs) [68], histone lactylation‐marked cis‐regulatory elements exhibit enhancer activity and are enriched near genes associated with cell adhesion and migration, such as *Zeb2* and *Sox10*. Furthermore, histone Kla peaks significantly overlap with transcription factors SOX9 and YAP/TEAD binding sites, suggesting that genes containing SOX or TEAD motifs are preferentially lactylated. Collectively, histone lactylation drives the expression of embryonic development‐related genes, linking embryonic cell metabolic states to the activation of GRNs.

Zygotic genome activation (ZGA) is a critical process in embryonic development, marking the transition from genomic silencing to active transcription. Li et al. [45] demonstrated that H3K18la is enriched at ZGA gene promoters to induce transcriptional activation, while lactate inhibition causes embryonic arrest at the two‐cell stage and downregulation of ZGA genes. This indicates that lactylation reshapes embryonic development, offering new strategies for improving embryo culture and assisted reproductive technologies.

## 4 Disease Association

Lactylation is closely associated with various diseases (Table 4). Therapeutic strategies targeting lactate or lactylation hold promising application prospects.

### 4.1 Mental and Neurological Diseases

Lactate, in addition to serving as an energy substrate, also participates in maintaining neuronal function and synaptic signalling. Therefore, abnormal lactate levels are closely associated with several disorders, including Alzheimer's disease, anxiety and stroke. These researches highlight the critical role of lactate‐lactylation in bridging ‘cell metabolism‐neuroregulation’.

Regarding Alzheimer's disease (AD), two positive feedback mechanisms that lead to a vicious cycle have been discovered: ‘PKM‐H4K12la‐PKM’ by Pan et al. [50] and ‘IDH3β‐lactate‐PAX6‐IDH3β’ by Wang et al. [49]. Pan RY found that inflammatory activation of microglia leads to metabolic reprogramming towards glycolysis and increased H4K12la, which promotes the expression of PKM. Inhibiting this positive feedback, such as *Pkm* knockout or PKM inhibitor shikonin or pyruvate dehydrogenase kinase (PDK) inhibitor DCA, can alleviate pathological changes and cognitive function in AD mice. Wang X discovered that the expression of the key enzyme of the TCA cycle, isocitrate dehydrogenase IDH3β, is reduced in AD, which leads to inhibited OXPHOS, accumulated lactate and increased histone Kla, including H3K18la, H4K8la and H4K12la. Confusingly, histone Kla usually upregulates target gene expression, which contradicts the reduction of IDH3β. Therefore, this might be related to a transcription regulatory factor. Further screening revealed that after inhibiting IDH3β, only the negative regulatory factor PAX6 increased. In summary, the reduction of IDH3β can increase histone Kla, promoting the expression of PAX6 and further inhibiting the expression of LDH3β. Thus, an ‘IDH3β‐lactate‐*Pax6*‐IDH3β’ positive feedback loop is formed. Overexpressing IDH3β or inhibiting PAX6 can improve neuronal complexity, dendritic spine number and behavioural or cognitive functions in AD.

In chronic restraint stress mouse model, Yan et al. [53] found that LDHA and lactylation were elevated in the prefrontal cortex. Lactylation omics revealed that differentially modified proteins were enriched in the ‘synaptic vesicle’ pathway, with the synaptic protein SNAP91‐K885la being the most significant. K855R point mutation reduced lactylation and led to decreased presynaptic vesicle density, abnormal synaptic structure, impaired neuronal excitatory transmission and ultimately exacerbated anxiety‐like behaviour. In summary, exercise can enhance synaptic structure formation and neuronal network activity by increasing SNAP91‐K885la, thereby exerting an anti‐stress effect.

In acute ischemic stroke, mitochondrial transfer from astrocytes to neurons is crucial for maintaining neuronal function and repairing damage. Zhou et al. [54] found that knockout of low‐density lipoprotein receptor‐related protein 1 (LRP1) leads to reduced mitochondrial transfer and increased lactate. LDH inhibitors resulted in reduced Kla increased extracellular mitochondrial and ATP levels. Conversely, lactate supplementation reduced mitochondrial ejection. Lactylation omics revealed the most significant upregulated Kla site following *Lrp1* knockout was ADP‐ribosylation factor 1 (ARF1)‐K73la. ARF1‐K73la inhibits mitochondrial transfer and exacerbates brain damage, while suppression of ARF1‐K73la reversed it. In the stroke mouse model, *Lrp1* knockdown mice exhibited larger infarct areas and worse neurological function. In summary, inhibiting LRP1 induced ARF1‐K73la can promote mitochondrial transport and improve stroke prognosis.

### 4.2 Ocular Diseases

A key pathological change in myopia is the increased differentiation of fibroblasts into myofibroblasts and reduced collagen, leading to scleral tissue remodelling. In *Hif‐1α* knockout myopia mice model, Lin et al. [46] discovered that highly expressed ENO1 exacerbates myopia by H3K18la. H3K18la activates the expression of *Notch1* in the insulin signalling pathway, thereby forming a ‘glycolysis—H3K18la—*Notch1*’ axis that induces myopia. The study suggests that reducing near work within 1 h after meals can prevent elevated insulin and scleral hypoxia that induce myopia.

In oxygen‐induced retinopathy, Wang et al. [55] found that microglia play a crucial role in neovascularisation through increased lactate and Kl. Lactylation omics focused on the transcription factor YY1‐K183la. YY1 itself has the function of regulating angiogenesis. The K183R mutation showed inhibited YY1's ability to promote angiogenesis in co‐cultured retinal microvascular endothelial cells. YY1 can also bind to the promoter of *Fgf2* to activate its transcription, ultimately promoting angiogenesis. In summary, inhibiting the ‘glycolysis‐YY1‐K183la‐FGF2’ axis is a potential therapeutic target in retinal neovascular diseases.

Autoimmune uveitis is characterised by abnormal differentiation of Th17 cells while glycolysis inhibitors or *Ldha* knockout can suppress it [69]. Fan et al. [56] found that lactate and Kla were also mostly elevated in Th17 cells rather than Th1 or Treg cells. The lactylation levels in Th17 cells induced from naïve CD4+ T cells were significantly higher than Th0 cells, and lactate could affect its differentiation. Lactylation omics focused on the transcription factor Ikzf1‐K164la. Ikzf1 is a highly conserved transcription factor involved in lymphocyte differentiation. K164R point mutation showed inhibited Th17 differentiation. Ikzf1‐K164 is also located within its DNA‐binding domain, suggesting that Ikzf1‐K164la may influence transcription. Cut&Tag results further indicated that after Th17 differentiation, CD4+ T cells exhibited significant enrichment of Ikzf1 binding peaks, including those of *Runx1*, *Tlr4*, *Il2* and *Il4*, which are closely related to the pro‐inflammatory response. This provides new insights into the pathogenesis of autoimmune uveitis and suggests that inhibiting Ikzf1‐K164la is a potential therapy target.

### 4.3 Cardiovascular Diseases

Cardiovascular dysfunction is usually accompanied by ischaemia and hypoxia, which provide necessary conditions for accumulated lactate and enhanced lactylation.

In atherosclerosis, the aging of vascular smooth muscle cells (VSMCs) is a critical pathological process. Li et al. [30] found that tumour necrosis factor receptor‐associated protein 1 (TRAP1) is upregulated in ruptured plaques and associated with cell aging. TRAP1 promotes the expression of phosphofructokinase 1 (PFK1) by reducing its ubiquitination, thereby upregulating glycolysis and H4K12la, which is enriched at the promoters of the senescence‐associated secretory phenotype (SASP). Overexpression of HDAC3 can reduce H4K12la and SASP, ultimately reversing the atherosclerosis phenotype. This study reveals the metabolic reprogramming axis ‘TRAP1‐HDAC3‐H4K12la’ where TRAP1 promotes the expression of SASP by inducing lactate accumulation and downregulating HDAC3 to increase H4K12la, ultimately leading to cell aging. Inhibitors of TRAP1, such as G‐TPP, and targeted chimeric degraders like PROTAC‐BP3 are new strategies for the treatment of atherosclerosis.

Vascular endothelial growth factor (VEGF) possesses a strong ability to promote endothelial cell proliferation, migration and angiogenesis. Fan et al. [28] discovered that VEGF increased the levels of LDHA and lactate in retinal microvascular endothelial cells. CUT‐Tag targeting the most significant upregulation histone Kla sites, H3K9la, showed *Nectin1*, *Tgfbr2*, *Abl1* and *Ptgfr* are enriched in pathways related to cell proliferation, adhesion junctions and angiogenesis. This indicates that H3K9la promotes VEGF‐induced angiogenesis. Notably, the enrichment of H3K9la at the promoter of the ‘eraser’ HDAC2 is downregulated. Overexpression of HDAC2 leads to a reduction in the expression of angiogenesis‐related genes and inhibits endothelial cell angiogenesis. This study suggests that ‘VEGF‐HDAC2‐H3K9la’ axis can promote angiogenesis, while disrupting this axis is a new direction for pathological neovascularisation.

Myosin and titin assemble into thick filaments, and their interaction is crucial for cardiac contraction. The head of myosin pulls the thin filaments in the presence of calcium ions and ATP, leading to myocardial contraction. In a heart failure mouse model, Zhang et al. [31] found lactylation decreased in the heart failure group, with the most significant decrease being α‐myosin heavy chain (α‐MHC)‐K1897la. α‐MHC‐K1897la promotes the interaction between α‐MHC and titin, while the K1897R point mutation not only reduced lactylation but also decreased its binding ability, leading to heart failure. Furthermore, its ‘writer’ is p300, and the ‘eraser’ is SIRT1. LDHA‐inhibited mice showed exacerbated heart failure, while sodium lactate or MCT4 inhibitors could upregulate α‐MHC‐K1897la and improve heart failure.

In myocardial infarction, early activation of macrophage repair signals is crucial. M1 macrophages promote plaque rupture by releasing MMP2 and MMP9, while M2 macrophages aid in tissue repair [70]. Wang et al. [19] discovered a significant increase in macrophage H3K18la in a myocardial infarction mouse model. CUT&Tag combined with RNA‐seq analysis revealed that H3K18la promotes the transcription of *Lrg1*, *Vegf‐a* and *Il‐10*, namely promoting M2 polarisation. H3K18la enhanced the anti‐inflammatory and angiogenic capabilities of macrophages, ultimately reversing pathological cardiac remodelling, promoting post‐infarction repair and improving cardiac function. Additionally, GCN5 (KAT2) was the ‘writer’.

### 4.4 Tumours

#### 4.4.1 Homologous Recombination Repair and Chemotherapy Resistance

Homologous recombination repair (HRR) utilises homologous DNA as a template to ensure the repaired sequence remains consistent, playing a crucial role in maintaining genomic stability. Most chemotherapy drugs work by damaging DNA to induce tumour cell death, and a significant part of the resistance arises from enhanced repairing damaged DNA ability, where lactylation exerts its effects.

MRE11 is significant in HRR, but its overactivation causes tumorigenesis. In breast cancer, Chen et al. [32] discovered that low expression of LDHA is associated with downregulated HRR, while sodium lactate can enhance it and induce chemotherapy resistance. Lactylation omics identified MRE11‐K673la and confirmed its ‘writer’ as CBP and ‘erasers’ as SIRT1/2. The MRE11‐K673R point mutation reduced its binding to DNA, thereby inhibiting HRR. LDHA or CBP inhibitors can enhance the cytotoxicity of cisplatin and olaparib. This study reveals that lactylation links tumour cell metabolism and DNA damage repair, serving as a key regulatory factor in HRR where chemotherapy combined with CBP or LDHA inhibitors could enhance sensitivity and provide a more efficient treatment.

BRCA1 and RAD51 are critical proteins in HRR, too. The formation of their foci marks the initiation and progression of repair, often used as indicators of DNA repair capability. In cisplatin‐resistant gastric cancer patients, Chen et al. [20] found lactylation was significantly elevated and led to chemotherapy resistance by enhancing HRR. NBS1 is involved in sensing and repairing DNA damage, with its K388 lactylated. NBS1 can form the MRE11‐RAD50‐NBS1 (MRN) trimer, thus enhancing HRR. NBS1‐K388R point mutation weakened the formation of foci and suppressed DNA repair. Additionally, a novel ‘writer’, TIP60 was identified. In summary, NBS1‐K388la enhances HRR by promoting the formation of the MRN complex, ultimately leading to chemotherapy resistance.

#### 4.4.2 Lactylation ‘Writers’ and Enhanced Tumour Malignancy

It is traditionally considered that p300/CBP are the ‘writers’ of lactylation, but recent studies have identified more. Interestingly, most of these newly identified ‘writers’ were discovered in cancer.

In exploring the ability of members of the MYST family to act as lactylation ‘writer’, Niu et al. [21] found that HBO1 exhibited the strongest activity. HBO1 knockout HeLa cells showed that 95 downregulated proteins enriched in nucleosome and DNA‐related pathways. Among them, H3K9la is the most significant histone site. Cell experiments demonstrated that H3K9la decreased after HBO1 knockout, accompanied by inhibited proliferation and invasion capabilities. Furthermore, elevated HBO1 and H3K9la were also found in clinical cervical cancer samples. These results suggest that HBO1, as a lactylation ‘writer’, activates the expression of tumour‐associated genes by mediating H3K9la, ultimately leading to tumour progression. Targeting HBO1 may provide new avenues for cervical cancer.

Given the structural similarity between lactate and alanine, and previous studies indicating that alany‐tRNA synthetase (AARS) functions in aminoacylation modification [71], Ju et al. hypothesised that AARS could also act as a lactylation ‘writer’ [25]. Further research indicated that AARS1 could convert lactate to a high‐energy intermediate in the presence of ATP, thereby catalysing lactylation. AARS1 could catalyse YAP‐K90la and TEAD1‐K108la, core components of the Hippo pathway, enhancing their interaction and ultimately exacerbating the malignancy of gastric cancer. Additionally, the ‘eraser’ is SIRT1. It is noteworthy that the function of AARS1 as a ‘writer’ differs from p300, as AARS1 directly utilises lactate while p300 relies on lactyl‐CoA.

In colorectal cancer, Xie et al. [22] discovered that lactylation is elevated and that the eukaryotic elongation factor 1 alpha 2 (eEF1A2) undergoes K408la. eEF1A2‐K408la promotes mRNA translation and protein synthesis, ultimately exacerbating tumour cell proliferation. Using immunoprecipitation combined with mass spectrometry to identify regulatory enzymes, KAT8, a lysine acetyltransferase with a MYST domain, exhibited the strongest affinity, suggesting that KAT8 is a potential lactylation ‘writer’. Further pathway enrichment showed that KAT8 is enriched in tumour‐related pathways such as translation, mitosis and the cell cycle. The knockout of KAT8 significantly inhibits tumour cell proliferation. In summary, KAT8 enhances the translation of tumour‐associated proteins by mediating eEF1A2‐K408la, ultimately leading to tumour progression. Targeting KAT8 represents a novel approach for the precise treatment of colorectal cancer.

#### 4.4.3 Lactylation and Tumour Metabolism

Warburg effect indicates that tumours can be considered as metabolic diseases. Studies have shown that Warburg effect is closely linked to the exacerbation of malignancy, drug resistance and cell cycle dysregulation. From a cellular metabolism perspective, activated glycolysis can lead to lactate accumulation and lactylation‐mediated metabolic reprogramming plays a key role in tumour.

Pancreatic ductal adenocarcinoma (PDAC) is one of the most invasive and lethal cancer types. Li et al. [29] found that H3K18la was elevated and negatively correlated with survival duration. Inhibiting glycolysis or knocking out LDHA could reduce H3K18la and suppress PDAC progression. CUT‐Tag and transcriptome sequencing targeting H3K18la revealed that TTK and BUB1B are targets. TTK and BUB1B are mitotic checkpoint regulators that are crucial for stabilising cell proliferation. However, high expression of TTK and BUB1 activated by H3K18la exacerbates the malignant proliferation. Additionally, BUB1 can enhance glycolysis and lactylation, while the knockout of TTK/BUB1B can reduce the ‘writer’ p300, suggesting a feedback regulation between TTK/BUB1B‐glycolysis‐lactylation. TTK can phosphorylate LDHA at Y239, which enhances the activity and then in turn increases H3K18la and further activates the expression of TTK and BUB1B. Thus, a positive feedback is formed: ‘LDHA‐H3K18la‐TTK/BUB1B‐LDHA’ Additionally, its ‘eraser’ was HDAC2. In summary, inhibiting TTK, BUB1B, LDHA, p300 or HDAC2 could disrupt this loop and offer a new strategy for treating the highly malignant PDAC.

In glioblastoma, Li et al. [57] discovered that patients with high aldehyde dehydrogenase (ALDH1A3) exhibit resistance to radiochemotherapy. ALDH1A3 can interact with PKM2 and enhance its tetramerisation, which ultimately leads to the accumulation of lactate. Moreover, the team focused on DNA damage repair‐related protein XRCC1 through enrichment analysis. XRCC1 has four lactylation sites, with K247la having the greatest functional impact. XRCC1‐K247la enhances its interaction with the nuclear transport protein importin‐α, promoting the nuclear localisation of XRCC1 and enhancing DNA repair, ultimately leading to resistance. Finally, the team developed a drug, D34‐919, that can precisely block the interaction between ALDH1A3 and PKM2, thus effectively inhibiting the growth of glioblastoma and increasing sensitivity to radiochemotherapy.

Adenylate kinase AK2 is a key enzyme that catalyses the production of ADP from ATP or AMP, and the TCGA database shows that AK2 is highly abundant in liver cancer. In the study of metabolic adaptation in liver cancer, Yang et al. [58] found that AK2‐K28la was significantly elevated and enriched in the ATP metabolic pathway. AK2‐K28la inhibits the activity of its phosphokinase, leading to energy metabolism disorders, ATP accumulation and tumour cell proliferation.

Fan et al. [52] found the glycolytic activity and Kla level of liver cancer stem cells were higher than ordinary liver cancer cells, especially aldolase ALDOA‐K230/322la and H3K56la. ALDOA‐K230/322la inhibits its binding to DDX17, prompting DDX17 to enter the nucleus and activate the expression of the stemness gene *Sox2*. H3K56la is enriched in the promoter of the stemness gene *Oct4* and induces its expression. In this way, ALDOA‐K230/322la and H3K56la jointly induce the stemness remodelling of liver cancer stem cells. In addition, the ‘writer’ of H3K56la is p300. Targeted inhibition of p300 or ALDOA is a new strategy to reverse the stemness of liver cancer stem cells.

### 4.5 Urinary System Diseases

Chronic kidney disease (CKD) is characterised by kidney fibrosis and persistent decline in renal function, where Wang et al. [51] discovered a significant increase of 6‐phosphofructo‐2‐kinase (PFKFB3) and H4K12la. PFKFB3 mainly localises in proximal tubular cells and is positively correlated with the severity of renal fibrosis. Knocking out *Pfkfb3* results in the suppression of renal collagen deposition and α‐smooth muscle actin, indicating alleviation of kidney fibrosis. In *Pfkfb3* knockout mice, renal fibrosis‐related genes and NF‐κB inflammatory pathway‐related genes were reduced. CUT&Tag showed that H4K12la was significantly enriched at the promoters of key NF‐κB signalling pathway genes such as *Ikbkb*, *Rela* and *Relb*. Thus, ‘PFKFB3‐H4K12la‐NF‐κB’ axis is formed. Targeting PFKFB3 could help alleviate renal inflammation and fibrosis, thereby slowing down or reversing CKD.

In lactate‐induced acute kidney injury [41], p300/CBP acts as ‘writers’ to catalyse HMGB1‐Kla and promotes the release of HMGB1 into the extracellular space via exosomes. Subsequently, HMGB1 drives the formation of neutrophil extracellular traps (NETs) by activating the TLR4/IL‐17 signalling pathway. Glucose analogue 2‐DG or p300/CBP inhibitors can alleviate kidney injury.

### 4.6 Fibrosis

Exercise is beneficial to health, but overtraining can exacerbate liver fibrosis. Liu et al. [59] found the accumulated lactate leads to SORBS3‐K479la, enhances its interaction with flotillin 1 and selectively promotes the sorting of F‐box protein 2 (FBXO2) into muscle‐derived small extracellular vesicles (SEVs). SEVs then activate hepatic stellate cells through the MCL1‐BAX/BAK signalling pathway, thereby exacerbating liver fibrosis. Inhibiting SORBS3‐K479la can alleviate liver fibrosis caused by overtraining.

In pulmonary fibrosis, Cui et al. [42] discovered that TGF‐β1 stimulated myofibroblasts had increased lactate. Bronchoalveolar lavage fluid from TGF‐β1‐ or bleomycin‐induced pulmonary fibrosis animal models also showed significantly increased lactate. In‐depth research revealed that metabolic reprogramming in myofibroblasts enhances glycolysis and lactylation, thereby promoting the expression of fibrosis‐related genes, such as *Vegf* and *Pdgfa*. Inhibition of the ‘writer’ P300 reduced the symptom.

### 4.7 Shock

Septic shock is characterised by a systolic blood pressure of less than 90 mmHg or a mean arterial pressure of less than 65 mmHg, accompanied by blood lactate higher than 2 mmol/L. ‘Lactate clock’ in macrophage promotes the shifting from pro‐inflammatory to anti‐inflammatory, which should play a positive role. However, Yang K [43] reported that macrophages take up extracellular lactate via MCT and induce lactylation of high mobility group box 1 protein (HMGB1). Subsequently, HMGB1 is released into the extracellular space via exosomes, reducing endothelial permeability, causing tissue damage and ultimately decreasing the survival rate in a septic mouse model. This suggests a negative effect of lactylation. Moreover, Chu et al. [47] reported that septic shock patients had a higher level of H3K18la and were strongly correlated with inflammatory mediators such as IL‐6 and procalcitonin. Therefore, H3K18la could serve as an auxiliary diagnostic marker for septic shock.

## 5 Discussion

### 5.1 Targeting Lactylation Is a Novel Direction for Disease Therapy

Targeting glycolytic enzymes, MCTs or regulatory enzymes has shown significant potential in disease therapy by precisely regulating the lactylation levels of specific proteins to alter their functions [72, 73]. However, it currently faces key challenges: (1) achieving site‐specific regulation without disrupting overall lactate metabolism; (2) overcoming the crosstalk effects within the PTMs network.

Targeting glycolytic enzymes, including pyruvate kinase (PKM), lactate dehydrogenase A (LDHA) and lactate oxidase (LOx), can affect lactate production and thereby intervene lactylation. Shikonin and compound 3 K can inhibit PKM and reduce the production of pyruvate, the lactate precursor, thereby (1) disrupting the H4K12la‐driven positive feedback in Alzheimer's disease [50], (2) improving chemotherapy resistance induced by XRCC1‐K247la [57]. Sodium oxalate (OXA) or FX11 can inhibit LDHA, directly reducing lactate production, thereby (1) blocking PCK‐K100la induced ferroptosis to alleviate ischemia–reperfusion injury [23], (2) inhibiting MRE11‐K673la and NBS1‐K388la induced DNA homologous repair recombination [20, 32], (3) reducing myopia induced by H3K18la [46], (4) inhibiting autophagy‐dependent survival of tumour cells [74]. Lactate oxidase (LOx) catalyses the conversion of lactate to pyruvate while generating reactive oxygen species (ROS), thus exerting a synergistic anti‐tumour effect [75].

Targeting monocarboxylate transporters (MCTs) can affect the transmembrane transport of lactate, thereby influencing lactate content and the local microenvironment [76]. MCT1 inhibitors, such as AZD3965 and silybin, can block the inward transport of lactate and reduce intracellular lactate content, thereby inhibiting cGAS‐K131/K156la to restore the antiviral immune response [27] and promote the transfer of astrocyte mitochondria to neurons to improve neurological function [54].

Regulatory enzymes including ‘writers’ and ‘erasers’ have been gradually identified, which show broad application prospects in disease therapy such as tumours and inflammations. Relevant information about the types, target sites, associated diseases, agonists and inhibitors of ‘writers’ and ‘erasers’ is presented (Table 3). It is worth noting that the agonists or inhibitors have problems such as insufficient specificity and the risk of off‐target effects. In addition, there are currently no effective specific agonists for HDACs.

### 5.2 Issues to Be Further Explored

As research progresses, more and more regulatory enzymes, including ‘writers’ and ‘erasers’, have been identified and show broad application prospects in diseases such as tumours and inflammation. The relevant content is listed in Table 3. It is worth noting that agonists or inhibitors targeting regulatory enzymes have problems such as insufficient specificity and the risk of off‐target effects. In addition, there are currently no effective specific agonists for HDACs.

Lactate is undergoing a transformation from being perceived solely as a metabolic waste product to being recognised as a signalling molecule involved in epigenetic regulation. A deeper understanding of lactylation is crucial for unfolding the complexity of cellular biology and pathophysiology. It also has the potential to open up new therapeutic approaches for related diseases, with targeted drugs against specific enzymes or modification sites being a promising direction for treatment strategies.

Although significant progress, there are still many unknowns to be explored. In terms of ultimate outcomes, does lactylation improve or worsen the body's response? Is lactylation a result or a cause of macrophage M2 polarisation? How are the promoters of M2 polarisation genes preferentially lactylated? The lack of widespread detection of lactyl‐CoA further complicates the understanding.

Regarding the process of lactylation, is it an inevitable consequence of lactate accumulation or a precisely regulated systemic process? If it is a consequence of lactate accumulation, what is the epigenetic threshold? If it is precisely regulated, what are the recognition mechanisms, the selectivity of lactylation sites and the regulatory factors involved in the modification process? Furthermore, what is the crosstalk and impact of lactylation on other PTMs?

## Author Contributions

**Tong Pan:** conceptualization (equal), data curation (equal), formal analysis (equal), investigation (equal), methodology (equal), project administration (equal), resources (equal), supervision (equal), visualization (equal), writing – original draft (equal), writing – review and editing (equal). **Can‐can Du:** data curation (equal), methodology (equal), project administration (equal), resources (equal), supervision (equal), writing – review and editing (equal). **Ying‐jian Zhang:** conceptualization (equal), data curation (equal), project administration (equal), resources (equal), supervision (equal), validation (equal), visualization (equal), writing – review and editing (equal). **Zhen‐long Liu:** conceptualization (equal), funding acquisition (equal), investigation (equal), project administration (equal), supervision (equal), validation (equal), visualization (equal), writing – review and editing (equal).

## Conflicts of Interest

The authors declare no conflicts of interest.

## Data Availability

Data sharing not applicable to this article as no datasets were generated or analysed during the current study.
